# Note on Chalybeate Therapy*Reprinted from the *Charlotte Medical Journal*, February, 1905.

**Published:** 1906-04

**Authors:** William Krauss

**Affiliations:** Memphis, Tenn.


### Abstracts and Selections.

### Note on Chalybeate Therapy.*

BY WILLIAM KRAUSS, PH. G., M. D., MEMPHIS, TENN.

There have been volumes written on chalybeate therapy and the controversies upon the absorbability of this or that form of iron have occupied the attention of clinicians for many years. Among

*Reprinted from the *Charlotte Medical Journal*, February, 1905.

the many preparations claiming the attention of clinicians, the one devised by Dr. Gude, chemist, of Leipzig, has received the endorsement of the ablest men on both continents for ten years. The writer has used it to the exclusion of all others, with the exception of a few disappointing experiments with others claiming equal merit, and has published two papers upon the subject.

It would seem that when one has used a certain medicament for years and with uniformly good results, and especially if this agent definitely eliminates the shortcomings of formerly employed products of the same elements, one is apt to take its effects as a matter of course, and further discussion would seem useless, or at least superfluous. When it happens, however, as is usually the case after a certain article has had a successful career, that many similar products strive to take the place of the original, generally resulting in disappointment to the user, it becomes a duty to take stock of the evidence in the case and see how far the confidence in the one and the distrust of the other is justified.

For this reason I have had compiled from separates and reprints some of the results of a few trustworthy observers.

1. Secondary Anemia.—H. D. Peterson (*Chicago Medical Recorder,* 1896), treated many cases, reporting 5 in detail, and says: "Pepto-Mangan is easily absorbed by the digestive tract without any disturbance of the same, is not injurious to the teeth, and produces no constipation."

Dr. J. W. Frazier, Vienna (*Therapeutische Monatshefte,* February, 1902), reports treating many cases, among which he made detailed studies upon 43 cases and summarizes: "It can be warmly recommended for extensive use in the treatment of anemic conditions."

Dr. Hugo Summa, St. Louis (*N. Y. Medical Journal,* 1895), recommends its use after tests in 34 cases, saying: "It is especially worth mentioning that no bad after-effects could be detected. In this connection I call special attention to the absence of constipation that could be traced back to the use of this preparation."

Dr. Sam'l Wolfe, Philadelphia, reports upon 50 cases observed during about four months, and concludes: "That Pepto-Mangan is a highly available preparation of iron, on account of its liquid form, pleasant to taste, non-corrosive action on the teeth and unirritating effect on the digestive organs, admitting thus of easy gradation of dose, easy administration to children and avoidance of unpleasant effects in all cases. That it is an efficient and rapid restorer of the normal quantity and quality of the blood, etc."

Dr. Fritz Euler-Rolle (*Wiener Klin. Rundschau,* March 29, 1903), mentions 14 cases of anemia, besides a number of cases of other diseases, in a very complete report in which he is pleased with its absorbability and (on account of the abundance of peptone it contains) its food value in delicate stomachs, and finds it free from all the objections usually urged against iron preparations, and its results prompt.

Dr. C. A. von Ramdohr (*N. Y. Medical Journal,* June 26, 1897), in connection with some gynecological cases, reports 7 cases of anemia, in which there was a rapid improvement.

Dr. H. P. Loomis, in a paper before the New York Academy of Medicine (June 18, 1893), reports a number of cases, 8 in detail, in whom there was a rapid increase in red cells and hemoglobin, and in most cases with no constipating effect.

Drs. Diago and Benitez, Superintendent and chief of Laboratory, Hospital No. 1, Havana, Cuba (*Progreso Medico,* Havana, April, 1902), report 6 cases in detail and summarize as follows: "We may say conscientiously that it is the best remedy we know of for the purpose, and that we do not hesitate to commend it to the profession, especially our confreres in Cuba, as an iron preparation that possesses all the advantages that can be demanded of such a remedy and none of the disadvantages that are characteristic of other iron preparations. We would especially emphasize also that Pepto-Mangan (Gude) is very pleasant to the taste, and is most easily taken by patients of all ages and with the most delicate digestions."

Dr. Juan Pablo Garcia, Havana (*La Revista Medica Cubana,* August 1, 1902), says: "I have had the opportunity of testing the efficiency of this preparation of iron in a large number of cases in both hospital and private practice, and have found it the most satisfactory iron compound that has come under my notice. It is not at all constipating, its taste is not astringent, so that it lacks the great disadvantages of most other iron compounds." He says further that it causes no disturbances of digestion and its therapeutic efficiency has been attested by the best clinicians throughout the world.

Dr. Louis J. Gravel, Physician-in-Chief to the Hotel Dieu, Montreal (*Buffalo Med. Journal.,* August, 1903), prefers the term dysemia, meaning "bad blood," reports 13 cases with blood examination, and says: "Comparing my results with Pepto-Mangan (Gude) with those obtained from other chalybeates of this class, I have been led to give it decided preference."

Drs. Chibas and George A. de Santos Saxe, of Columbus Hospital, New York (*Int. Jour. Surg.*, June, 1903), report 40 cases, with tabulated results and complete bibliographic review (which has facilitated this compilation very much), and say they have used it in the hospital for over two years in anemic convalescents with uniformly satisfactory results. "In no case did constipation, nausea, headache, or digestive difficulties follow its administration."

Dr. Hermann Metall, Assistant Physician at the General Polyclinic, Vienna (*Med. Chir. Centralblatt,* January, 1902), reports 23 cases of anemia consequent upon a variety of conditions, of which 12 showed a normal hemoglobin per cent after fourteen days, 5 after three weeks, and 5 after a month. He concludes that it is "a reliable and valuable blood-building remedy, which can be recommended for general use in appropriate cases."

Anemia Consequent Upon Special Conditions.—Dr. Mateo M. Guillén, at Randall's Island Children's Hospital, gives a tabulated report of 32 cases of infantile anemia with very elaborate blood chart, including some very desperate cases of cachexias, and says: "In no case did we have to suspend treatment on account of any untoward influence on the delicate organisms of sick infants." Three cases classed as hopeless made a complete recovery. The paper is very instructive.

Dr. J. M. Freiser (*vide supra*) also reports great success in infantile anemia.

Dr. J. K. Bauduy (*St. Louis Medical Review,* February 26, 1898), reports upon a number of cases of neurasthenia, 12 of them in detail, with blood examinations by Dr. Carl Fisch, and says: " * * * the results, however, were indeed a surprise to myself, for the concomitant deranging sequelæ were so slight that but in very few instances in my extensive utilization * * * was I obliged to discontinue it. * * * this particular remedy, I am now convinced, will prove a great boon to the patient and the physician. * * * of course, we do not consider the remedy applicable to cases of lithemic neurasthenia, nor in any manner a specific in any variety of neurasthenia."

Dr. Ludwig Pohl, City Physician, Vienna (*Aertzl. Centr. Anzeiger,* September, 20, 1899), has used Pepto-Mangan (Gude) in over 100 cases of chlorosis, anemia, neurasthenia, hysteria, and malarial cachexia, and says: "I have previously mentioned that it may be positively assumed that Pepto-Mangan (Gude) stimulates the hemopoietic organs to increased activity. * * *

Decided amelioration in the leukemic state, arrest of the process in severe cases for a long time, reduction of the glandular swellings, improvement in the relation between the red and white corpuscles, were noted by me in several cases."

Dr. J. S. Perekhan (*Chicago Clinical Recorder*, 1896), reports a number of cases, 6 in detail; Dr. C. A. von Ramdohr (*vide ref. supra*), 12 cases in detail; and Dr. Gellhorn, at Mackenroth's Clinic, Berlin (*Therap. Monatshefte*, 1897), mentions 60 cases, some in detail, all gynecological patients with a variety of conditions, some post-operative, and all testify to the improved blood findings, Dr. Gellhorn concluding as follows: "I feel justified in asserting that in my therapeutic trials with Pepto-Mangan I obtained all that can be rationally demanded."

In surgical conditions calling for improvement in the blood, Drs. Stuart McGuire (*Vir. Med. Semi-Monthly*), 20 cases, and George G. Van Schaick (*N. Y. Med. Journal*, June 2, 1900), 50 cases, report favorable result. The latter concludes: "We have in such a preparation as Pepto-Mangan (Gude) a means of obtaining good results with a certainty that is almost mathematical, and without any of the distressing symptoms so frequently following the use of the inorganic iron preparations."

Dr. H. Edwin Lewis, Burlington, Vt. (*Vt. Med. Monthly*), Dr. Ed. C. Hill, Denver, Dr. W. O. Davis (*N. Y. Int. Journal Surg.*, September, 1902), together with some of the authors already mentioned call especial attention to the value of the preparation in irregular menstruation, sterility and other sexual anemias in women.

Dr. Karl von Ruck (*N. Y. Medical Journal*) finds in Pepto-Mangan (Gude) the best preparation in the anemia of tuberculosis, being more efficient and more easily borne; he had used it in 70 cases, 12 being reported in detail, in some of which comparative tests were made with other iron preparations. Other observers also mention it in tubercular anemia.

Cachexia finds especial mention by Fritz Euler Rolle, Pohl, and Fasano, the latter professor at Royal University, Naples (*Arch. Int. di Med. e Chir.*, March, 1899), discusses iron medication in detail, calling special attention to the chemistry of the subject and reports having treated primary anemia, 20 cases; chlorosis, 25 cases; malarial anemia, 7 cases; tubercular anemia, 8 cases; uterine diseases, 11 cases; scrofulosis, 12 cases; rachitis, 10 cases; convalescents of exhausting diseases, 15; total, 107 cases. He says: "To recapitulate, Pepto-Mangan (Gude) not only deserves the place it has already acquired in therapeutics, but it merits even greater recognition, because all clinicians ought to make use of it in pathological processes in which the object is to restore to its normal condition the altered quality of the blood."

After this array of evidence it is only necessary to add that since this constitutes about all the clinical literature upon which our knowledge of the subject is based, it is rather significant that it was all done upon this particular product. In no instance is it claimed that the preparation is as good as something else, but it is simply set forth by the observers that this was the preparation used and that it met these indications in the manner described. Since there is no official preparation that meets these requirements the manufacturers of Pepto-Mangan (Gude) deserve all the credit which the product has earned for them.

